# Determining the impact of postoperative complications in neurosurgery based on simulated longitudinal smartphone app-based assessment

**DOI:** 10.1007/s00701-021-04967-0

**Published:** 2021-08-21

**Authors:** Lion D. Comfort, Marian C. Neidert, Oliver Bozinov, Luca Regli, Martin N. Stienen

**Affiliations:** 1 Department of Neurosurgery, University Hospital Zurich, Frauenklinikstrasse 10, 8091 Zurich, Switzerland; 2 Clinical Neuroscience Center, University of Zurich, Zurich, Switzerland; 3 Department of Neurosurgery, Cantonal Hospital St. Gallen, St. Gallen, Switzerland

**Keywords:** Functional impairment, Patient-reported outcome measures, Health-related quality of life, Classification of surgical complications, Smartphone, mHealth

## Abstract

**Background:**

Complications after neurosurgical operations can have severe impact on patient well-being, which is poorly reflected by current grading systems. The objective of this work was to develop and conduct a feasibility study of a new smartphone application that allows for the longitudinal assessment of postoperative well-being and complications.

**Methods:**

We developed a smartphone application “Post OP Tracker” according to requirements from clinical experience and tested it on simulated patients. Participants received regular notifications through the app, inquiring them about their well-being and complications that had to be answered according to their assigned scenarios. After a 12-week period, subjects answered a questionnaire about the app’s functionality, user-friendliness, and acceptability.

**Results:**

A total of 13 participants (mean age 34.8, range 24–68 years, 4 (30.8%) female) volunteered in this feasibility study. Most of them had a professional background in either health care or software development. All participants downloaded, installed, and applied the app for an average of 12.9 weeks. On a scale of 1 (worst) to 4 (best), the app was rated on average 3.6 in overall satisfaction and 3.8 in acceptance. The design achieved a somewhat favorable score of 3.1. One participant (7.7%) reported major technical issues. The gathered patient data can be used to graphically display the simulated outcome and assess the impact of postoperative complications.

**Conclusions:**

This study suggests the feasibility to longitudinally gather postoperative data on subjective well-being through a smartphone application. Among potential patients, our application indicated to be functional, user-friendly, and well accepted. Using this app-based approach, further studies will enable us to classify postoperative complications according to their impact on the patient’s well-being.

**Supplementary Information:**

The online version contains supplementary material available at 10.1007/s00701-021-04967-0.

## Introduction

If complications would not exist, neurosurgical operations could be offered to patients without hesitation and patients would not need to feel uneasy about an upcoming procedure. Complications occur after about 5–50% of neurosurgical operations, however, depending on their definition and way of recording. They have a varying impact on a patient’s subjective well-being, functional status, and quality of life. Depending on a particular complication’s type and severity this impact can range from mild to severe, it can be temporary or permanent and it might require non-operative or operative action to resolve. Despite their particular importance, complications are not yet well recorded and classified.

Currently, postoperative complications are commonly graded according to the Clavien-Dindo grade (CDG) [3, 5]. This grading system indicates, which kind of treatment is required to deal with a particular complication. With this treatment-centered classification system, however, the impact a complication may have on the patient’s well-being is neglected [23]. Moreover, the current methods to follow-up patients after neurosurgery are not optimal, as they are static and rely on snapshots at fixed points in time, while in reality, the clinical course may vary substantially over time [2]. With our current follow-up methods, there is no mechanism to detect unfavorable clinical courses before the situation exacerbates.

Due to the already widespread and currently increasing availability of modern smartphone technology, managing postoperative outcomes via mobile applications is the future [2, 15]. Mobile health applications have the potential to significantly improve patient lives [25]. It has been shown that telemedicine for post-discharge surgical care is safe, effective, and acceptable to both patients and health care providers [13]. Mobile health applications for postoperative monitoring have been successfully tested in the fields of gastrointestinal, gynecological, and orthopedic surgery besides neurosurgery [12, 22, 27]. In the field of spine surgery, in particular, applications for patient management and outcome assessment proved to be successful and well appreciated by patients [8, 14, 19, 26]. However, none of the aforementioned studies and applications laid a special focus on the impact of postoperative complications on the patient’s well-being.

We recently developed a smartphone application, in order to longitudinally capture our patient’s subjective well-being and record postoperative complications after neurosurgery. Besides recording outcome data on the patient level, another goal of this app is to help determine the impact of a particular complication on a patient’s outcome. This feasibility study was conducted to assess, whether our app is operational, user-friendly, and well accepted among potential applicants.

## Methods and materials

### App overview

#### Development

We developed the first prototype of the app, named “Post OP Tracker”, focusing on the Google Android platform. Post OP Tracker was developed on the environment Android Studio® (Mountain View, CA, USA) [10] using the Oracle Java ® (Austin, TX, USA) programming language [11].

#### First use

It is anticipated that patients are informed about the app in the preoperative period during outpatient clinics. The app should be downloaded and installed during clinics either by the patient him-/herself, supported by the physician or hospital staff if needed.

**Fig. 1:**
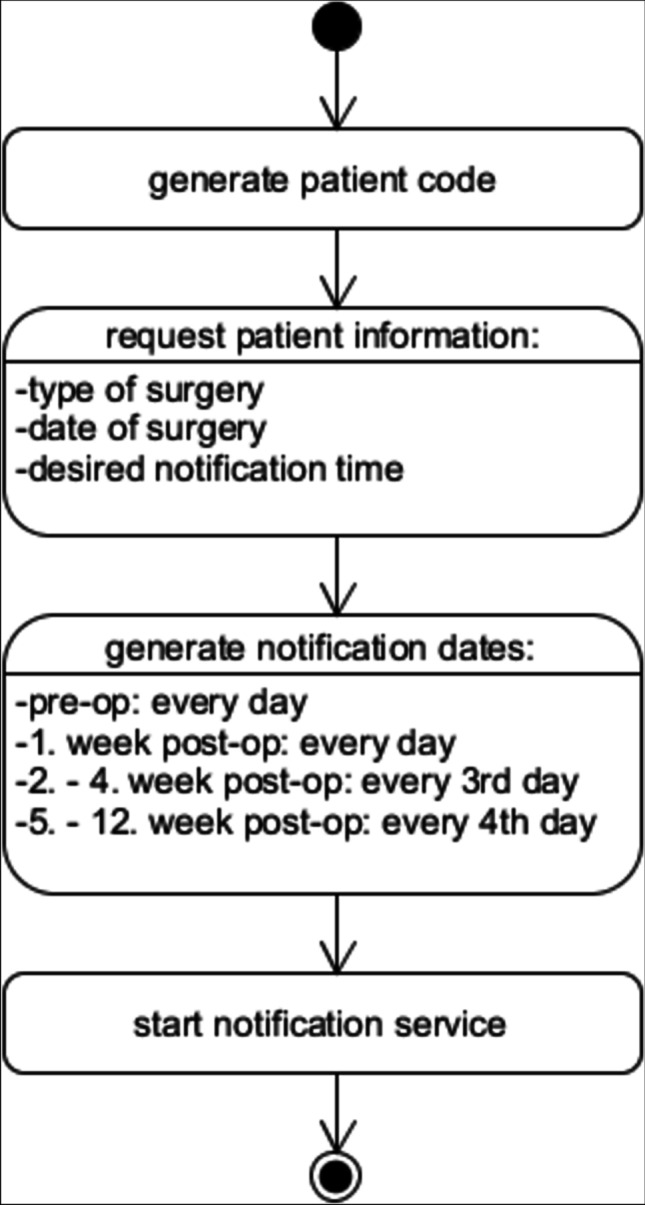
Activity diagram of the first use

**Fig. 2:**
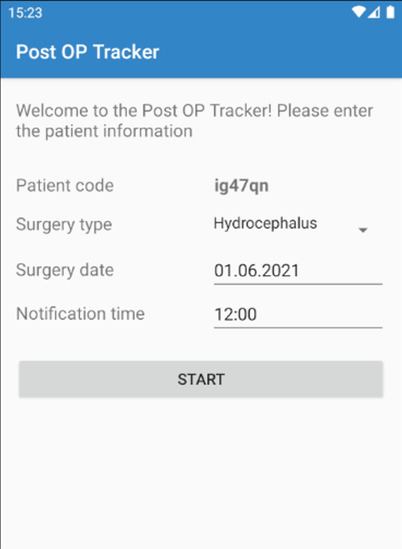
Screenshot of the first use. Screenshots of the app were translated to English for the purpose of this paper

Upon first use, the Post OP Tracker app generates a code for each individual patient, an alphanumeric string with six characters (Figs. 1 and 2). This code is used to keep patients anonymous throughout the data collection period and prevent privacy concerns, but to be able and identify each patient at a later stage, at the time of data upload to the web server. The app then requests the type of surgery being performed, using a drop-down list. In the pilot phase and for purpose of this feasibility analysis, we only programmed four representative choices covering essential areas in neurosurgery (hydrocephalus, brain tumor, disc prolapse, and brain aneurysm), but this list can easily be extended. The date of anticipated surgery is added, as well.

#### Notification service

The central function of the Post OP Tracker app is the notification service, which is why the patient can choose the notification time according to his/her preference. At this pre-set notification time, patients receive a push notification [9], requesting him/her to enter a pre-defined set of variables (see “Patient entry” below).

Before and in the first week after the operation, patients receive this notification on a daily basis, in order to establish a solid preoperative “baseline evaluation” and to measure patient well-being closely within the most dynamic early postoperative period. Of note, on the day of the operation, no notification is displayed. As the frequency of complications decreases and the functional status becomes more stable the longer out of surgery, the frequency of notifications is lowered from daily to every 3rd day from the 2nd to the 4th postoperative week, then to every 4th day from the 5th through the 12th postoperative week. This timing for postoperative notifications was chosen to optimize compliance and ensure patient adherence (patients will not be “overloaded” with notifications), while capturing the status closely in the most fluctuating “early postoperative period.” The notification service is designed to run in the background at all times and to pop up at the pre-set time. If patients do not respond to the pop-up immediately, the notification will stay in the notification bar.

After the 12th postoperative week has passed and the last notification has appeared, the data inquiry ceases, and no more inputs are requested from the participant for this feasibility study.

#### Patient entry

**Fig. 3:**
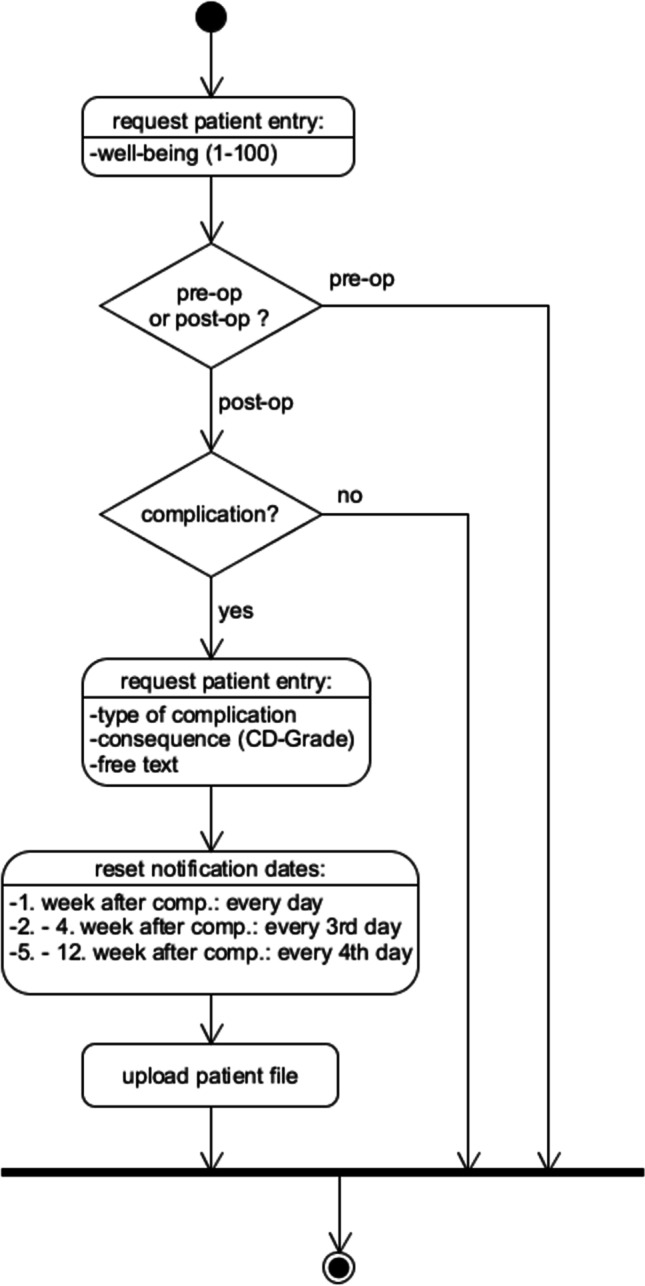
Activity diagram of the patient entry; CD, Clavien-Dindo grade of postoperative complications

**Fig. 4:**
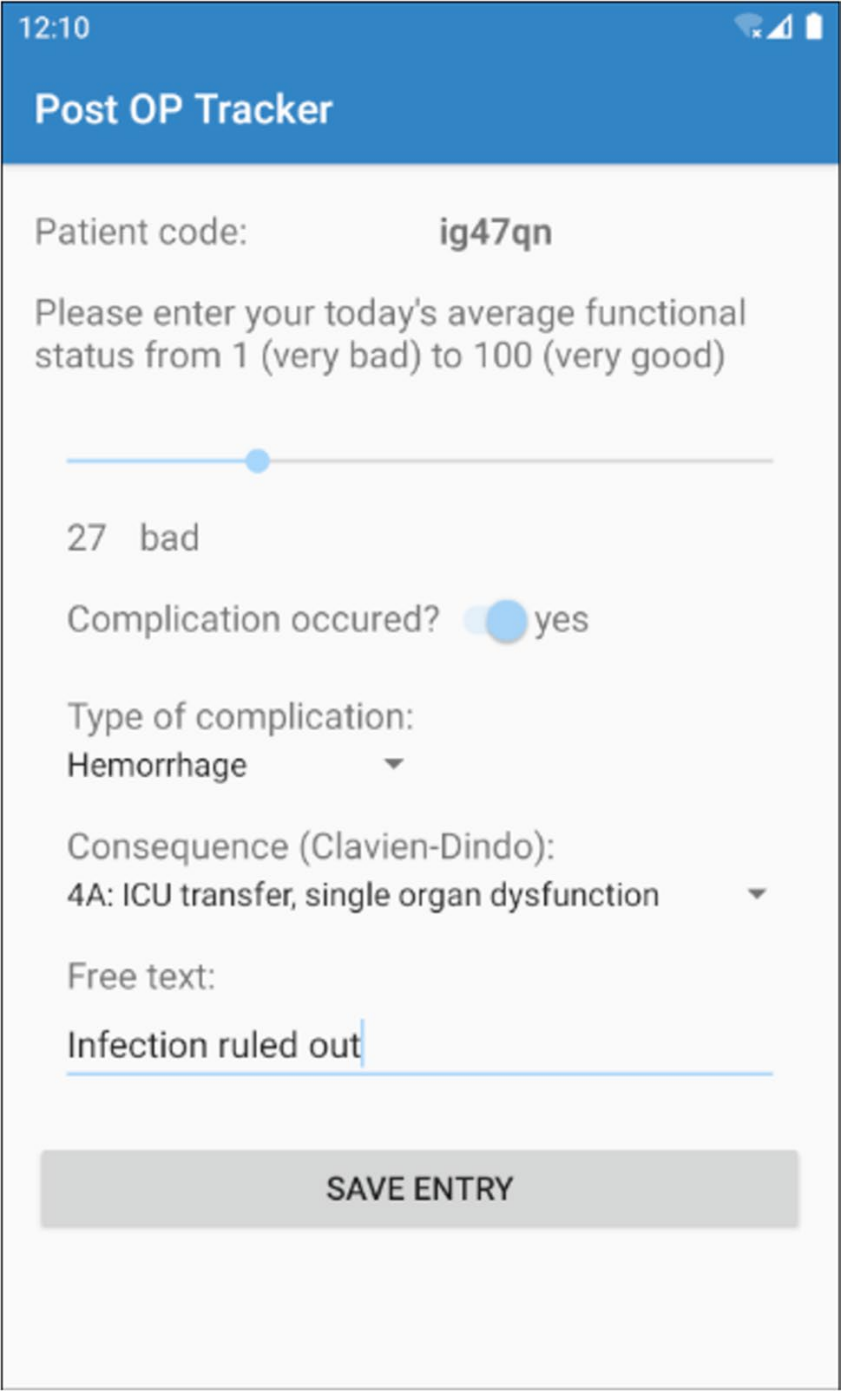
Screenshot of the postoperative patient entry with the complication “hemorrhage” and the Clavien-Dindo grade 4A as consequence

On each day of data entry, a patient receives a push notification at the pre-set time, requesting him or her to enter his/her subjective functional status from 1 (worst) to 100 (best), using a slider bar (Figs. 3 and 4). If the entry occurs postoperatively, the patient can also indicate, whether a complication occurred, using a switch button. If no complication occurred, the patient has completed the day’s requirement and the data is saved locally on the smartphone device.

If a complication occurs, further input elements appear on the app’s screen, which can be entered by the patient or a health care professional. The user first chooses the type of complication using a drop-down list (e.g., hemorrhage, seizure, infection, etc.) and classifies the complication according to the Clavien-Dindo grading scale (CDG; Table 1) [5]. The user has the possibility to enter a string of free text, if desired (e.g., “purulent drainage and increasing tenderness of the wound”).

**Table 1:** Clavien-Dindo grading scale (CDG) [5]

Grades	Definition
Grade I	Any deviation from the normal postoperative course without the need for pharmacological treatment or surgical, endoscopic, and radiological interventions
	Allowed therapeutic regimens are as follows: drugs as antiemetics, antipyretics, analgesics, diuretics and electrolytes, and physiotherapy. This grade also includes wound infections opened at the bedside
Grade II	Requiring pharmacological treatment with drugs other than such allowed for grade I complications
	Blood transfusions and total parenteral nutrition are also included
Grade III	Requiring surgical, endoscopic, or radiological intervention
- IIIa	Intervention not under general anesthesia
- IIIb	Intervention under general anesthesia
Grade IV	Life-threatening complication (including CNS complications)* requiring IC/ICU-management
- IVa	Single organ dysfunction (including dialysis)
- IVb	Multi-organ dysfunction
Grade V	Death of a patient

^*^Brain hemorrhage, ischemic stroke, subarachnoid bleeding, but excluding transient ischemic attacks; *CNS*, central nervous system; *IC*, intermediate care; *ICU*, intensive care unit

Whenever a complication occurs, the notification dates are reset, treating the complication as if it were a new operation and thus increasing the interval of post-complication patient inputs. The rationale is to again increase the frequency of capturing the patient’s well-being, as we anticipate the patient status is less stable/more variable compared to a complication-free postoperative course. This reset, however, does not affect the static 12-week runtime of the app. Additionally, all of the patient’s data is uploaded to a web server whenever a complication is entered.

#### Patient data

All of the patient’s entered data is stored locally on the patient’s smartphone, using the JSON format [7]. Those JSON files contain the patient’s code, the performed surgery, the date of the surgery, and all of the entries the patient made upon receiving notifications. The entries consist of the date of the entry, the patient’s functional status (1–100) and, if applicable, the complication together with grading and the string of free text.

Data uploads from the patient’s smartphone to the web server are conducted on several occasions:

1. Mandatorily upon entering a complication,
2. automatically on the last day of each month, and
3. manually by the patient at each time point, should he/she wish to do that.
4. Finally, at the end of the app’s runtime (12th week postoperative), the patient is prompted to upload his/her data.

The JSON patient files are uploaded to a password-protected web server, using the File Transfer Protocol [18]. The uploaded patient data can be accessed online by the responsible health care professional, who at this stage can also verify if the data is complete and the patient entries occurred regularly. Using Microsoft Excel® (Redmond, WA, USA), the patient data can be imported and displayed in a comprehensible manner.

### Study design

This feasibility study was designed as a prospective, monocentric observational study. Participants were healthy individuals without prior disease or injury of the brain or spine. Since this study enrolled no patients, no approval by an institutional review board was required. We planned to recruit 15 subjects for this analysis.

### Participant identification

We recruited volunteers by contacting coworkers, friends, and family members. Most of them either had a professional background in health care or in software development. The participants received a handout, containing general information about the app and the study aims, an installation manual, and a user guide.

To be included, subjects had to fulfill the following criteria:

1. Owning an Android smartphone
2. Willingness to input data on a daily basis for 12 weeks
3. Willingness to fill out a questionnaire at the end of the runtime.

Prior diseases or injuries of the brain or spine were our exclusion criteria.

For each participant, we mapped out a scenario, defining the type and date of surgery and describing the postoperative outcome. Subjects were required to simulate that outcome as realistically as they could. We equally distributed favorable, ordinary, and unfavorable postoperative outcomes among the participants. Those simulating an unfavorable outcome were the only participants required to enter postoperative complications. For each entered complication, we specified a realistic description, timeframe (days of onset and duration), and impact on the subjective well-being. We detailed the complication’s impact on the subjective well-being with descriptions concerning health-related quality of life and activities of daily living.

### Participant questionnaire and rating scale

At the end of the runtime (12 weeks postoperative), we asked the participants to fill out a questionnaire, using Google Forms® (Mountain View, CA, USA). Subjects were asked to give basic demographic information and their patient code, which linked them to their anonymous data uploads.

We asked questions concerning the participant’s overall satisfaction and their adherence to regular patient inputs. Further inquiries concerned temporal effort of patient inputs, usability, design, and the willingness to use the Post OP Tracker app as a patient and as a health care professional (concerning only health care professionals).

For each of those questions, we gave the choice of four possible answers on a Likert scale, ranging from.

1. not user-friendly
2. a little user-friendly
3. somewhat user-friendly
4. very user-friendly.

Additionally, we included questions concerning the technical operability of the app. We wanted to know if the notifications appeared regularly and at the desired time, and if there were any technical problems during the runtime of the study. Technical problems as well as a general feedback could be given using free text answers.

The complete questionnaire is given in Online Resource 1.

## Results

We recruited 15 volunteers, of which 13 (86.7%) used the Post OP Tracker app until the end of the runtime and filled out the questionnaire (Table 2). One volunteer ceased the participation because of personal reasons, another one because of misunderstandings regarding the daily patient inputs. All other participants downloaded, installed, and applied the app for an average of 12.9 weeks. The mean age was 34.8 years (range 24–68). Four participants were female (30.8%), nine were male (69.2%). Five subjects reported a professional background in health care (38.5%); four had a background in software development (30.8%). Five participants simulated an unfavorable outcome (38.5%) and thus entered postoperative complications.

**Table 2:** Participant baseline characteristics

Mean age, range	34.8 (24–68)
Sex	
Female	4 (30.8%)
Male	9 (69.2%)
Profession	
Health care	5 (38.5%)
Software development	4 (30.8%)
Other	4 (30.8%)
Simulated surgery type	
Hydrocephalus	3 (23.1%)
Brain tumor	3 (23.1%)
Disc prolapse	3 (23.1%)
Aneurysm	4 (30.8%)
Simulated outcome	
Favorable	4 (30.8%)
Ordinary	4 (30.8%)
Unfavorable, with complications	5 (38.5%)

### Participant satisfaction

Participants reported an average satisfaction of 3.6/4. The app design was the lowest-rated entity on the questionnaire with an average score of 3.1/4. The willingness to use the Post OP Tracker app, both as a patient or as a health care professional, was rated highest with a score of 3.8/4 (Table 3).

**Table 3:** Participant satisfaction

Entity	Mean score (range)
Overall satisfaction	3.6 (3–4)
Adherence to regular patient inputs	3.6 (2–4)
Temporal effort of patient inputs	3.7 (3–4)
General usability of the app	3.6 (3–4)
Design of the app	3.1 (2–4)
Willingness to use app as health care professional	3.8 (3–4)
Willingness to use app as patient	3.8 (3–4)

### Technical reports

Table 4 includes inquiries concerning technical aspects of the app. An important aspect of Post OP Tracker is the regularity of the notifications, requesting patient entry. Six users received the notifications regularly at the desired time (46.2%); one user did not (7.7%). The remaining six users who answered “other” (46.2%) were asked to further explain their answers. Some of those users reported a slight delay of a couple of minutes. Other users reported a 1-h delay after the change from daylight saving time to standard time. Indeed, this subject was overlooked by us when developing the app and concerned only users who started their runtime before the day of time change.

**Table 4:** Technical reports

Did the notifications appear regularly at the desired time?	
Yes	6 (46.2%)
No	1 (7.7%)
Other	6 (46.2%)
Did you encounter any technical issues?	
No	10 (76.9%)
Yes, minor	2 (15.4%)
Yes, major	1 (7.7%)

Ten users reported no technical issues (76.9%); two reported minor issues (15.4%). One user reported a missing notification at the end of the 12-week runtime, which would have requested him to make his last entry. Another user explained some uncertainties concerning the patient upload. The user who reported a major technical issue was the same person who answered the question concerning regularity of notifications with no. This person, who installed our app on a Huawei phone, was shown no notifications at all. It is a known issue that due to the strict settings concerning power savings, Huawei phones terminate background processes [28], in our case, the service showing the requesting patient entry notification.

## Discussion

Our results suggest that the Post OP Tracker app in its current state is operational and can be used to longitudinally collect patient’s inputs on their subjective well-being and postoperative complications. Overall, our volunteers/simulated patients were very satisfied with the Post OP Tracker with regard to usability and willingness to using it in a clinical setting. The design of the app received only somewhat favorable ratings and some technical issues were reported, which help us now to improve the app before applying it to real patients within a clinical study.

After these experiences gained throughout the feasibility analysis, we consider it highly advisable to further apply the Post OP Tracker to real neurosurgery patients. Recent studies have shown a high level of interest in and access to smartphone apps aiding postoperative recovery in neurosurgery patients [14, 17, 26]. Generally, enabling patients in monitoring their treatment can have a positive impact on patient’s engagement and outcomes [1, 15]. Observing the quality of postoperative recovery via mobile applications appears to be feasible to both patients and surgeons [2, 24]. Further development and implementation should focus on usability of technology and personal contact with the study personnel, as those factors are key contributors to maximizing participant engagement in mobile health studies [6].

### Simulated outcome examples

We would like to illustrate how the Post OP Tracker app’s graphical results can be analyzed and interpreted.

**Fig. 5:**
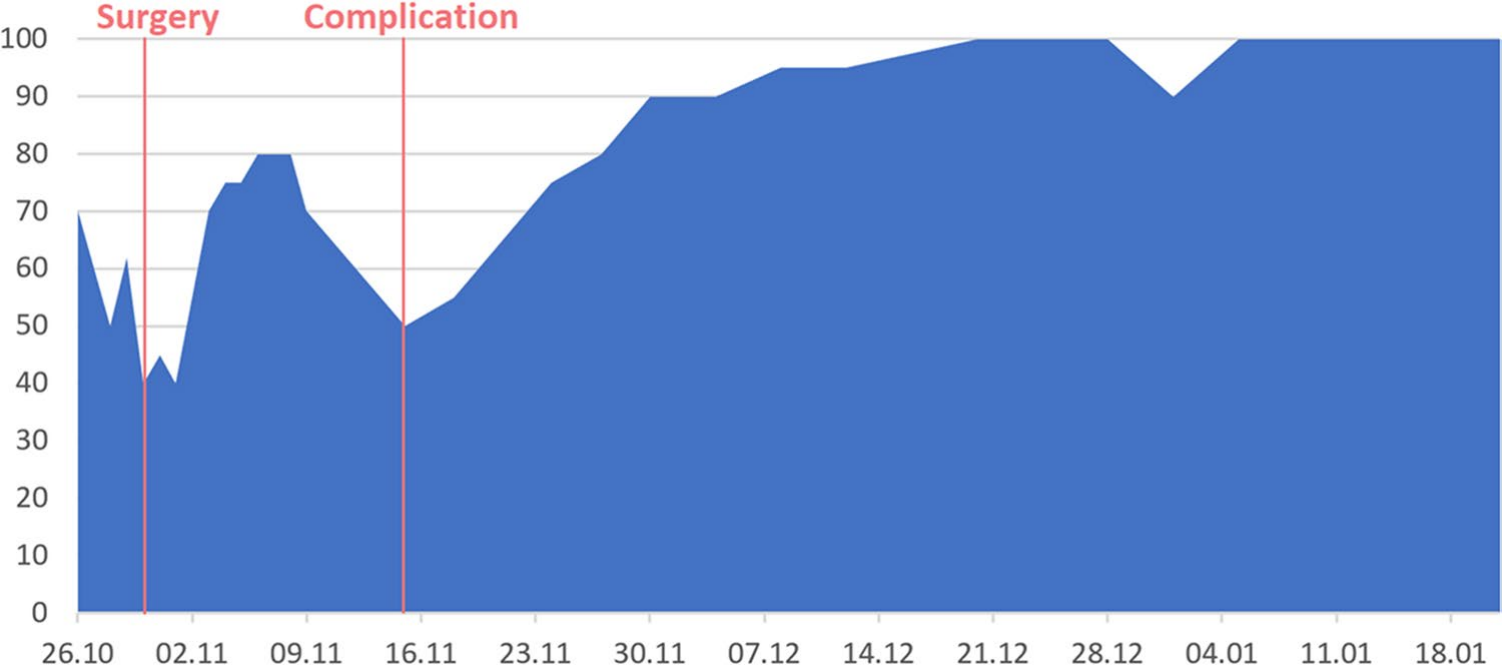
Simulated outcome of a hydrocephalus operation with a complication

Figure 5 shows an area graph of the simulated outcome of a participant. The *y*-axis shows the patient’s subjective well-being on a scale of 1 to 100; the *x*-axis the day of entry, ranging from 1 week before the operation to 12 weeks after the operation. This participant was tasked to simulate receiving a ventriculoperitoneal shunt operation for normal pressure hydrocephalus with a singular complication arising around 2 weeks after the surgery. The first decline in well-being marks the days preceding and immediately following the day of surgery, which may indicate anxiety besides progressive signs of hydrocephalus before and incisional pain from the wound after surgery. After this, the well-being increases as the patient recovers sharply but declines somewhat until the pre-defined complication arises. The complication was defined to be a generalized epileptic seizure that had to be treated with anticonvulsants (Clavien-Dindo grade II) in this scenario. The participant then simulated recovery from the complication in the following weeks.

**Fig. 6:**
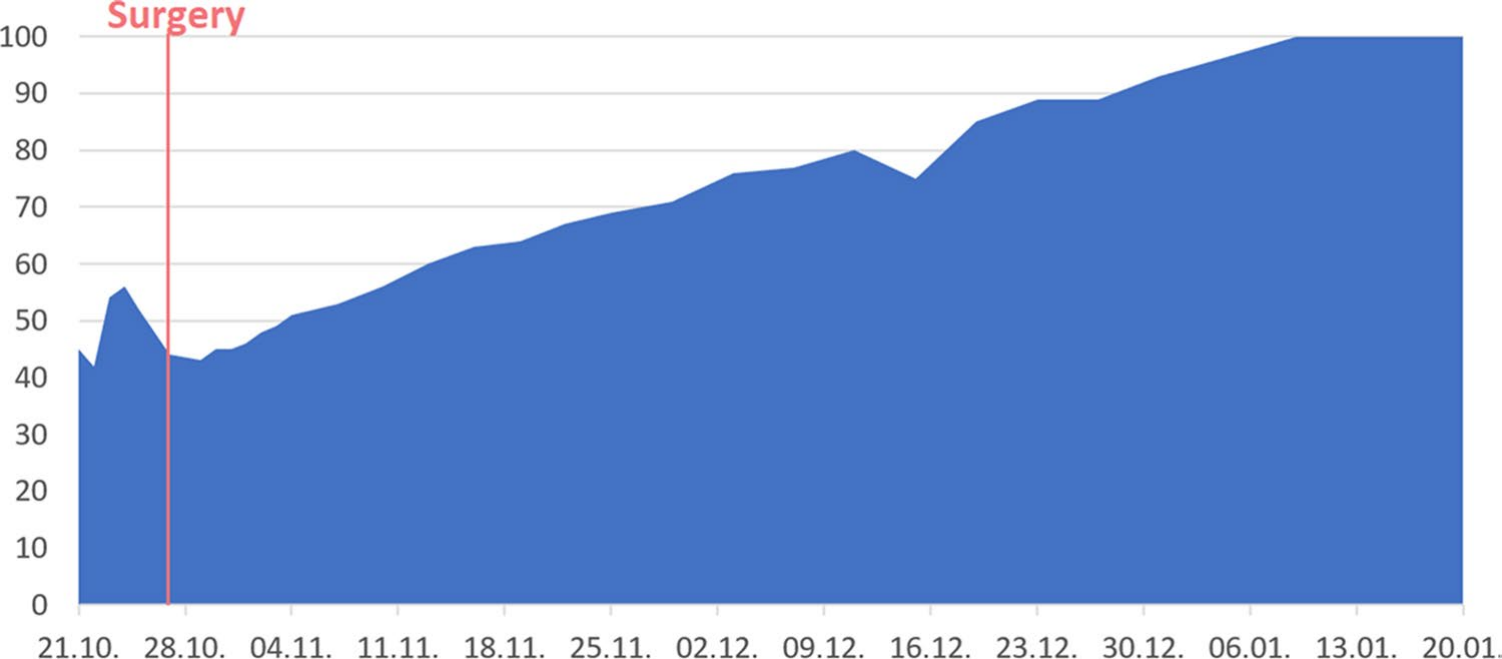
Simulated outcome of a hydrocephalus operation without complications

Figure 6 shows the outcome of another participant undergoing the same surgery, however without any postoperative complications. Note how this participant chose to simulate a slower recovery than the participant in Fig. 5. However, in this case vignette, no complication occurred and, hence, no decline in the subjective well-being was recorded, as opposed to Fig. 5.

### Future outlook: application of the app in neurosurgery

These two case vignettes contain individual and hence highly variable datasets. By gathering data from a high number of real patients after neurosurgery through the app, however, we could determine an “average normal postoperative course” for each type of surgical procedure without a postoperative complication.

In a second step, we could compare the outcome and postoperative course of patients who experienced any type of complication from those with an uneventful postoperative course. The difference between the “normal” and “abnormal” postoperative course could be calculated and its magnitude would be a measure of complication severity. This approach would take into consideration the impact of a complication on the patient’s well-being, other than the current classification systems.

**Fig. 7:**
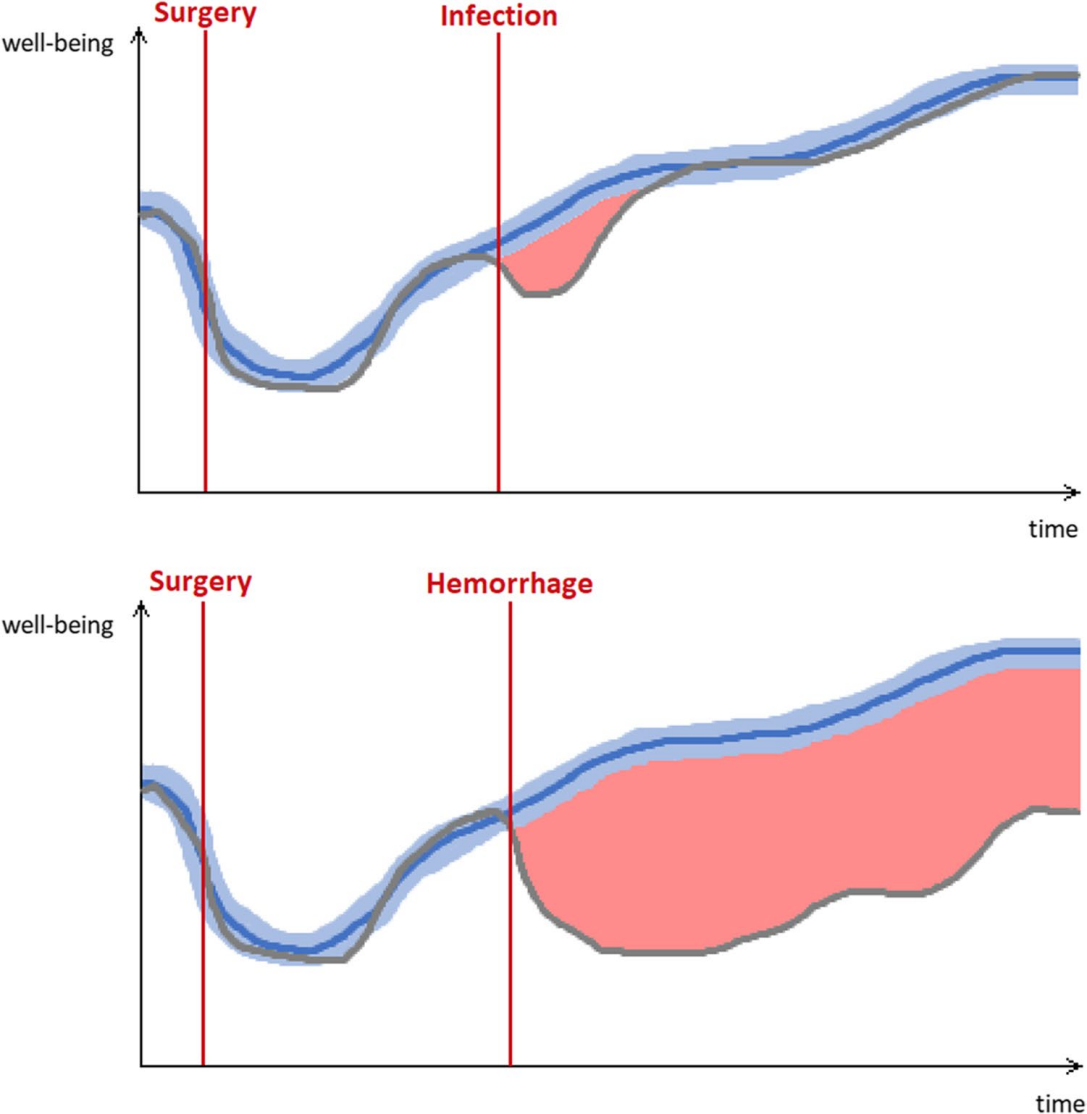
Two exemplary outcomes. The blue line indicates the average postoperative outcome of a shunt surgery with 95% CI. The gray line displays the described outcome for the imaginary patients. While the patient with a urinary infection reaches the average functional status again quickly, the patient suffering from intracerebral hemorrhage will likely never recover back to normal

For example, an imaginary 57-year-old female undergoing a shunt procedure for normal pressure hydrocephalus experiencing a postoperative urinary infection on day 5 (treated with antibiotics and total resolution 1 week later) will be classified as a CDG II type of complication. The same patient, now experiencing a small intraparenchymatous hemorrhage in the left basal ganglia, resulting in permanent hemiparesis and aphasia, will be classified as CDG I type of complication (as no treatment is required). The Post OP Tracker app will be able and differentiate between the severity of these complications better, as outlined in Fig. 7. Please note that the area between the average postoperative course and the individual postoperative course in patients with a postoperative complication can be used as an indicator of complication severity (marked in red color). With collected data with the help of this app, we could re-define and validate a new classification system for postoperative complications by ranking complications according to their impact on the patient’s subjective well-being.

A word of caution is required with regards to app-based data collection in patients that experience any type of complication resulting in cognitive decline or aphasia. Both complication sequelae would interfere with a patient’s ability to respond to the Post OP Tracker app’s pop-ups. Once we apply this app to real patients within the framework of an observational study, there are two options how to deal with this situation: First, if in doubt, the ability of a patient to continue with the study could be evaluated with cognitive screening tools, such as the Montreal Cognitive Assessment (MoCA) [16], a brief and effective tool in neurosurgery patients [20]. Patients with presumably permanent significant deficits will no longer be required to perform data entries and hence exit the study. However, as patients may recover over time, data entry could be temporarily halted and again continued after clinical improvement. Second, a patient’s next-of-kin might be approached to provide the missing data points during the time when the patient is unable to respond him-/herself. Both ways of dealing with this situation are sub-optimal from a scientific point of view but are pragmatic solutions that serve to estimate the impact of a given complication as accurately as possible.

### Strength and limitations

We are aware that our current sample is limited (*n* = 13). The possibility of selection bias can also not be denied by the authors, since health care professionals and software developers are not necessarily reflective of the average neurosurgery patient population. Simulated “patients” were included in this feasibility study, who made their entries according to our pre-defined scenarios and their best knowledge. This, of course, is only an estimate of how patients, who experience real postoperative complications, will accept the app and classify outcome, especially during the onset and progression of neurological symptoms (e.g., postoperative headache, prolonged nausea or dizziness, neurological impairment, repetitive seizures …). However, we decided that this simplified approach is an ideal setting for testing a prototype app, without bothering patients and avoiding ethical issues. Technical operability has been established, as only one participant reported major technical issues, but this important feedback is helpful to improve the app, before applying it to patients. We chose the classic visual analog scale [21] as a way of representing the subjective functional status in order to minimize the temporal effort of patient inputs and thus ensuring patient adherence. However, more sophisticated patient-reported outcome measures (PROMs) of pain, disability, or quality of life could easily be implemented in the future. The exclusive availability of the Post OP Tracker app on Google Android phones is another limitation of our study. Further development should aim at an additional Apple iOS version, considering the iPhone’s market share of 43% in Switzerland [4]. A big strength is our active involvement in the development of our app, which allowed us to conserve financial resources and tailor Post OP Tracker precisely to our needs.

## Conclusions

This study suggests the feasibility of developing a new smartphone application for longitudinal postoperative monitoring in neurosurgery. Pilot-testing the app in healthy individuals/ “simulated patients”, we recorded high satisfaction rates, which are encouraging to take on the next step and applying the app to real patients. Implementing this big data approach, future studies can define detailed “normal” postoperative courses after specific neurosurgical procedure types. Postoperative complications can then be classified as deviation from a normal course, with complication severity defined according to its magnitude.

## Supplementary Information

Below is the link to the electronic supplementary material.

Supplementary file1 (PDF 398 KB)

## Abbreviations

App: Application
CDG: Clavien-Dindo grade
CI: Confidence interval
CNS: Central nervous system
IC: Intermediate care
ICU: Intensive care unit
JSON: JavaScript object notation
OP: Operation
PROMs: Patient-reported outcome measures
