# Supplementary material for: Determining the impact of postoperative complications in neurosurgery based on simulated longitudinal smartphone app-based assessment
Source: Acta Neurochir (Wien). 2021 Aug 21;164(1):207–17. doi: 10.1007/s00701-021-04967-0 (PMC8761146; doi:10.1007/s00701-021-04967-0)
Supplement: Supplementary file 1 — Supplementary file1 (PDF 398 KB) [file 701_2021_4967_MOESM1_ESM.pdf]

Description:
A Google Forms ® print of the questionnaire we sent to our participants. Questions were translated to English for the purpose of this publication.

Article title: Determining the impact of postoperative complications in neurosurgery based on longitudinal smartphone app-based assessment

Journal: Acta Neurochirurgica

Authors: Comfort LD (1,2), Neidert MC (3), Bozinov O (3), Regli L (1,2), Stienen MN (3)

Affiliations:
(1) Department of Neurosurgery, University Hospital Zurich, Zurich, Switzerland
(2) Clinical Neuroscience Center, University of Zurich, Zurich, Switzerland
(3) Department of Neurosurgery, Cantonal Hospital St. Gallen, St. Gallen, Switzerland

Corresponding Author:
Lion D. Comfort, Department of Neurosurgery, University Hospital Zurich
comfortlion@gmail.com

Post OP Tracker Questionnaire

*Required

Email adress: *

Your answer

What is your patient code?

Your answer

How old are you? *

Your answer

What is your gender? *

- ○ male
- ○ female

What is your profession? *

Your answer

How good do you rate the app? *

- ○ very good
- ○ good
- ○ medium
- ○ bad

How regularly did you enter your patient inputs? *

- always
- mostly
- rarely
- never

How high was the temporal effort of entering the patient inputs? *

- low, < 5 seconds
- medium, 5 - 10 seconds
- high, 10 - 20 seconds
- very high, > 20 seconds

How user-friendly did you perceive the app in general? *

- very user-friendly
- somewhat user-friendly
- little user-friendly
- not user-friendly

Did the notification appear regularly and at the desired time? *

- yes
- no
- Other:

Did you experience any technical issues when using the app? *

- no
- yes, minor
- yes, major

Please specify the technical issues if you answered the last question with 'yes':

Your answer

How good do you rate the design of the app? *

- ◯ very good
- ◯ good
- ◯ moderate
- ◯ not good

For health care professionals: can you imagine your patients using this app in the future?

- ◯ definitely
- ◯ possibly
- ◯ rather not
- ◯ never

Can you imagine using this app as a patient? *

- ○ definitely
- ○ possibly
- ○ rather not
- ○ never

Do you have further suggestions or remarks? If yes, please enter them here:

Your answer

Submit

Never submit passwords through Google Forms.

This content is neither created nor endorsed by Google. Report Abuse - Terms of Service - Privacy Policy

Google Forms
